# The Association between Metformin and the Cancer-Specific Mortality Rate in Nasopharyngeal Cancer Patients: Real-World Evidence

**DOI:** 10.3390/curroncol30040298

**Published:** 2023-03-30

**Authors:** Yen Hsu, Chung Y. Hsu, Yung-Shuo Kao

**Affiliations:** 1 Department of Family Medicine, Changhua Christian Hospital, Changhua 500, Taiwan; 2 Graduate Institute of Biomedical Sciences, China Medical University, Taichung 404, Taiwan; 3 Department of Radiation Oncology, Taoyuan General Hospital, Ministry of Health and Welfare, Taoyuan 330, Taiwan

**Keywords:** nasopharyngeal cancer, Metformin, cohort study, survival analysis

## Abstract

Objectives: Nasopharyngeal cancer is a common cancer in East and South Asia. The radiotherapy and chemotherapy regimen has advanced in recent years. However, many patients still suffer from local recurrence and distant metastasis; thus, identifying medication that can be combined with standard treatment to improve the treatment outcomes in nasopharyngeal cancer patients is an unmet need. Methods: We included nasopharyngeal cancer patients from the Taiwan National Health Insurance Database (NHIRD). The primary endpoint was set as the cancer-specific mortality rate. Metformin cohorts and non-Metformin cohorts were matched by sex, age, and the year of the index date. Propensity score matching with a ratio of 1:1 was applied. Results: A total of 6078 subjects were included in the study, with 3039 patients in each group. Male participants outnumbered female participants. Most of the patients were aged 50 to 64; the mean age was 60.4 ± 10.4 years in Metformin non-users, and that of Metformin users was 59.9 ± 10.5 years. Metformin users had a lower risk of death due to nasopharyngeal cancer (adjusted HR = 0.80; 95% CI = 0.71, 0.90) than controls. Conclusions: We concluded that Metformin might be effective at reducing the cancer-specific mortality rate in nasopharyngeal cancer patients. Further randomized control trials should be completed.

## 1. Introduction

Nasopharyngeal cancer (NPC) is a common cancer in East and South Asia [1]. In Taiwan, the diabetic population rises year on year. According to the IDF Diabetes Atlas, in 2021, the number of people with diabetes in Taiwan was estimated at 2,450,000. At the same time, it was reported that over 140 million people aged 20–79 years had diabetes in China [2]. Both of these two are major areas where nasopharyngeal cancer occurs.

In previous research, the interaction between diabetes mellitus (DM), nasopharyngeal cancer, and metformin has been discussed. Guo et al. indicated that, compared with people without DM, the diabetic population is at a lower risk of nasopharyngeal cancer, but this has little effect on survival [3]. In addition, Zhang et al. suggested that metformin has an impact on cancer risk. Decreased frequency of head and neck cancer, lung cancer, liver cancer, pancreatic cancer, colorectal cancer, bladder cancer, and prostate cancer was reported in metformin users [4,5,6,7]. Tseng also revealed that NPC incidence was reduced with metformin use, even in subgroup analyses based on factors such as age, gender, and patients with or without nephropathy and liver diseases [8]. These statements boosted the evidence of metformin preventing cancer.

So far, the main treatments for nasopharyngeal cancer are radiotherapy and chemotherapy. The radiotherapy and chemotherapy regimen has advanced in recent years. However, many patients still suffer from local recurrence and distant metastasis; thus, identifying medications that can be combined with standard treatment to improve the treatment outcomes in nasopharyngeal cancer patients is an unmet need.

As mentioned above, there have been many studies confirming the benefit of metformin in the cancer population. In some clinical settings, metformin is already prescribed to enhance the response to cancer therapy. Some evidence indicates that metformin can prevent multiple cancers in vivo or in vitro, or in observational studies [9,10]. Thus, we are interested in the role of diabetes medications in nasopharyngeal cancer.

Metformin is the first-line treatment for type II diabetes mellitus. This medicine has been widely used for decades and is still the main option for diabetes mellitus treatment nowadays. The mechanism of type II diabetes is related to insufficient insulin secretion and insulin resistance. It acts on the liver and reduces glucose formation by inhibiting gluconeogenesis and lipogenesis by 5’-adenosine monophosphate (AMP)-activated protein kinase (AMPK). It also involves muscle and fat tissue to enhance insulin sensitivity and promote glucose uptake in the bloodstream.

Other agents used in diabetes, such as insulin secretagogues, including sulfonylureas and glinides, bind to sulfonylurea receptors on the pancreas beta cell and stimulate insulin release. In addition, α-glucosidase inhibitors act on the proximal intestine to suppress the degradation of complex carbohydrates to monosaccharides and glucose. Thiazolidinedione activates PPAR-γ to promote the insulin sensitivity of muscle, adipose tissue, and the liver. DPP-4 inhibitors and GLP-1 can raise the concentration of incretins, which stimulates insulin release and reduces glucagon production. SGLT2 inhibitors block the resorption of glucose from proximal convoluted tubules and accelerate the excretion of glucose into the urine.

As a previous study conducted in Taiwan showed, metformin is effective at reducing the risk of nasopharyngeal cancer in diabetes patients [3,4,8]. Some basic research has also shown that metformin could inhibit the growth and proliferation of nasopharyngeal cancer cells, reverse the drug resistance of cisplatin, and make cells more radiosensitive [11,12]. Shi also reported metformin could facilitate NPC cell apoptosis. In a time and concentration-dependent manner, metformin alone significantly inhibited the activity of CNE1 cells, which are NPC cell lines. Furthermore, the combination of cisplatin with metformin exhibited stronger inhibition of NPC cell activity, migration, and invasion [1,13].

As a result, we would like to know whether metformin could reduce the cancer-specific mortality rate in nasopharyngeal cancer patients. After a literature search, we did not find any research investigating this issue. We aim to use the national population-based cohort of Taiwan to gather real-world evidence on this important topic.

## 2. Methods

### 2.1. Data Source

Taiwan launched the National Health Insurance (NHI) program in 1996, which included more than 99% of the 23 million Taiwanese. The data, including the demographic characteristics of the insured and records of admission and discharge, medicine, and treatment from all medical care settings, are stored in the Taiwan National Health Insurance Database (NHIRD). In order to identify patients with nasopharyngeal cancer, we used the linked registry of the Registry for Catastrophic Illness Patient Database (RCIPD), which was derived from the NHIRD of Taiwan. The diagnostic codes of patients in the RCIPD and NHIRD were based on the International Classification of Diseases, Ninth and Tenth Revision, Clinical Modification (ICD-9-CM and ICD-10-CM). This study was approved by the Research Ethics Committee of China medical university and hospital (CMUH109-REC2-031(CR-2)).

### 2.2. Study Population

Patients diagnosed with nasopharyngeal cancer (ICD-9: 147, ICD-10: C11) between 2000 and 2017 were the study subjects of this retrospective cohort study. We divided the patients into 2 groups: subjects taking Metformin or not. The index date of Metformin users was defined as the first prescription date of Metformin after patients were diagnosed with nasopharyngeal cancer, and that of Metformin non-users was set as a random date after the diagnosis of nasopharyngeal cancer. Two cohorts were matched by sex, age (in 5-year intervals) and the year of the index date. Propensity score matching with a ratio of 1:1 was applied. Excluded from this study were those patients under 20 years old, those who died due to nasopharyngeal cancer before the index date, and those with missing data on sex and age.

### 2.3. Main Outcome and Covariates

The study outcome of interest was death due to nasopharyngeal cancer. The related comorbidities were hypertension (ICD-9: 401–405, ICD-10: I10–I15), hyperlipidemia (ICD-9: 272, ICD-10: E78), chronic obstructive pulmonary disease (ICD-9: 491, 492, 496, ICD-10: J41, J43, J44), chronic kidney disease (ICD-9: 585, ICD-10: N18), and heart failure (ICD-9: 428, ICD-10: I50) that occurred before the index date. Moreover, we considered not only the related drugs, including Sulphonylurea, Thiazolidinediones, AGI, Insulin, DPP4, and Meglitinides but also treatments for cancer, such as radiation therapy and chemotherapy.

### 2.4. Statistical Analysis

A chi-squared test was used to assess the baseline categorical variables between the 2 cohorts, and the difference in mean age was estimated by a Student’s *t*-test. The incidence rate was calculated with a unit of 1000 person-years. The unadjusted and multivariable-adjusted hazard ratios (HR and aHR), with corresponding 95% confidence intervals (CIs) for the risk between 2 cohorts, were analyzed by a Cox proportional hazard regression model. We measured the cumulative incidence curves between Metformin users and comparison cohorts using the Kaplan–Meier method and compared the difference with a log-rank test. All statistical analyses were presented using SAS software, version 9.4, and plots were plotted by R software, version 4.0. The statistical significance level was set to *p* < 0.05.

## 3. Results

A total of 6078 subjects were included in the study, with 3039 patients in each group. As Table 1 shows, male participants outnumbered female participants. Most of the patients were aged 50 to 64; the mean age was 60.4 ± 10.4 years in Metformin non-users, and that of Metformin users was 59.9 ± 10.5 years. Compared to the controls, there were more Metformin users who took the related medications, and the most common medication in the former was insulin (Metformin non-user: 60.6% vs. Metformin user: 76.5%) and that in the latter was Sulphonylurea (Metformin non-user: 44.3% vs. Metformin user: 77.0%). The distributions of the comorbidities and treatment for the 2 groups were significantly different, including hypertension (Metformin non-user: 68.3% vs. Metformin user: 66.0%), hyperlipidemia (Metformin non-user: 64.3% vs. Metformin user: 55.2%), chronic kidney disease (Metformin non-user: 5.36% vs. Metformin user: 2.20%), heart failure (Metformin non-user: 5.23% vs. Metformin user: 4.15%), radiation therapy (Metformin non-user: 85.8% vs. Metformin user: 76.8%), and chemotherapy (Metformin non-user: 71.6% vs. Metformin user: 63.4%).

**Figure 1:**
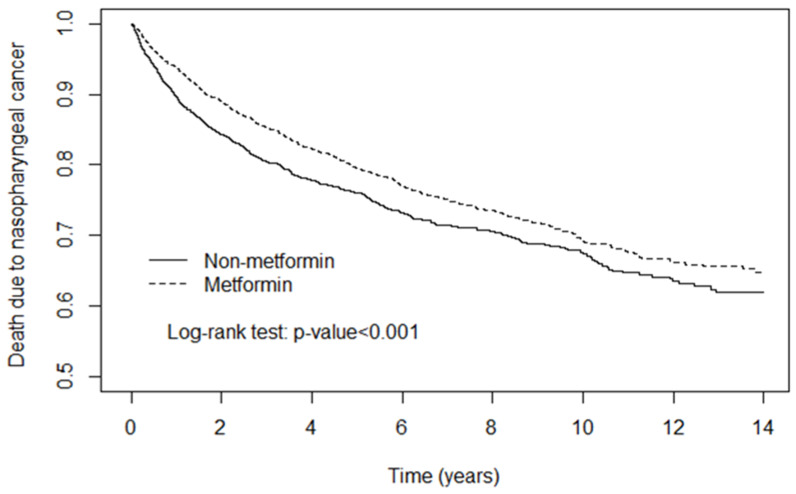
Kaplan–Meier model revealed that the cumulative incidence of death due to nasopharyngeal cancer was lower in the Metformin group than that in the non-Metformin group at the end of the follow-up period (*p* < 0.001).

Table 2 illustrates the risk of death due to nasopharyngeal cancer among the two groups. Overall, after adjustment for age, sex, comorbidities, medications, and treatment, Metformin users had a lower risk of death due to nasopharyngeal cancer (adjusted HR = 0.80; 95% CI = 0.71, 0.90) than controls. In addition, among male patients, the risk of death in Metformin users was lower than in Metformin non-users (adjusted HR = 0.79; 95% CI = 0.69, 0.91). For patients aged 50 and up, the Metformin cohort had a lower hazard ratio compared to subjects not taking Metformin (50–64: adjusted HR = 0.72; 95% CI = 0.61, 0.86; over 65: adjusted HR = 0.80; 95% CI = 0.66, 0.96). No matter which drugs or treatment, the risk of death due to nasopharyngeal cancer was lower in the Metformin group than in the control group. Moreover, the risk of death in Metformin users was 0.73-fold lower than in patients not using Metformin (95% CI = 0.64, 0.83) among patients with comorbidities. In the Figure 1, the cancer-specific survival rate of patients is represented on the vertical axis, with a comparison between those taking metformin and those not taking it. On the horizontal axis, two groups of individuals are shown based on their capacity to survive in definite time. As Figure 1 indicates, the cumulative incidence of death due to nasopharyngeal cancer in Metformin users was significantly lower than that in non-Metformin users.

As shown in Table 3, the hazard ratio of death due to nasopharyngeal cancer was higher for males (adjusted HR = 1.45; 95% CI = 1.27, 1.65) than for females. The risk of death increased by 1.03-fold for each additional year of age of the patient. Compared with subjects not taking related drugs such as AGI and DPP4, those taking these drugs had a significantly lower risk of death (AGI: adjusted HR = 0.83; 95% CI = 0.71, 0.97; DPP4: adjusted HR = 0.64; 95% CI = 0.56, 0.74). In contrast, patients taking insulin (adjusted HR = 2.16; 95% CI = 1.87, 2.50) or having chemotherapy (adjusted HR = 2.03; 95% CI = 1.73, 2.38) had a relatively higher hazard ratio than those not undergoing these treatments. As for comorbidities, the risk of death due to nasopharyngeal cancer for subjects also diagnosed with hypertension (adjusted HR = 1.16; 95% CI = 1.02, 1.31), chronic kidney disease (adjusted HR = 1.58; 95% CI = 1.22, 2.03), or heart failure (adjusted HR = 1.68; 95% CI = 1.32, 2.13) was higher than for subjects without these accompanying diagnoses.

By stratifying taking Metformin and AGI or not into different groups, the risk of death in patients using Metformin but not AGI (adjusted HR = 0.86; 95% CI = 0.75, 0.98) was lower than in those not taking either. Furthermore, patients using both were at a significantly lower risk of death due to nasopharyngeal cancer (adjusted HR = 0.62; 95% CI = 0.50, 0.76).

## 4. Discussion

To our knowledge, this cohort study is the first population-based study to investigate the effect of Metformin on reducing the cancer-specific mortality rate in nasopharyngeal cancer patients. The strength of this study is that we included a large number of patients in this cohort study, which forms a strong evidence base.

In Table 1, we see that the Metformin group was less likely to receive radiotherapy and chemotherapy. This may be because the patient not receiving radiotherapy or chemotherapy was at stage 4 or less willing to receive treatment. Another interesting phenomenon is that the Metformin group was less likely to have hyperlipidemia and chronic kidney disease. These treatments and comorbidities were adjusted in further analysis.

From Table 2, we note that metformin is effective at reducing the cancer-specific mortality rate. In this study, we adjusted age, sex, comorbidities, medication and treatment, and the incidence rate of death shows a significant difference between metformin users and non-users. The results verify the hypothesis that Metformin is a potential factor in improving the prognosis of nasopharyngeal cancer (adjusted HR = 0.80; 95% CI = 0.71, 0.90). The Kaplan–Meier curve also confirms this result (*p* < 0.001). We conducted a further subgroup analysis and found that Metformin was effective only in male patients. There had been a previous study showing that the glycemic control effect of Metformin is different in males and females [14]. Also, there is basic research showing that Metformin is more effective at reducing carcinogenesis in male mice [15].

As people get older, mortality from nasopharyngeal cancer increases (adjusted HR = 1.03; 95% CI = 1.02, 1.03). However, the benefit of metformin in terms of lowering the risk of death from nasopharyngeal cancer was only found in patients older than 50. In the meantime, there was no difference in patients aged under 50 years. We, therefore, suggest that Metformin be given to patients over 50 years.

To determine the effect of metformin, we compared different combined regimens of antihyperglycemic agents. Our study revealed that people who did not take Sulphonylurea showed no difference in NPC death rate, no matter whether they used Metformin or not. On the contrary, the combination of Metformin and Sulphonylurea use led to a positive outcome. Regarding the Thiazolidinediones, insulin, DDP4 inhibitor, or Meglitinides user group, adding metformin could bring about an advantage, decreasing the mortality rate compared to patients not using Metformin. These results are possibly because of the better glycemic control involved with the use of Metformin. In addition, among patients who received either radiation therapy or chemotherapy, the group who added Metformin after a diagnosis of NPC demonstrated a significant reduction in mortality.

A multivariate analysis revealed that oral hypoglycemic agents such as AGI and DPP4 inhibitors reduce nasopharyngeal cancer mortality. AGI was also shown to be able to reduce colorectal cancer incidence [16]. Basic research has also demonstrated that acarbose is able to impede renal cancer growth [17]. However, research into this effect in nasopharyngeal cancer is still lacking, so we cannot conclude that AGI is effective at reducing the cancer-specific mortality rate in nasopharyngeal cancer patients. DPP4 inhibitors were shown to be effective, improving the survival rate in prostate cancer patients [18]. In basic research, dipeptidyl peptidase IV (DPP4), a protein, was identified as a cancer-related item [19]. Thus, the DPP-4 inhibitor can be an effective regimen for suppressing nasopharyngeal cancer cells. Further basic research should be conducted using DPP4 inhibitors and nasopharyngeal cancer cell lines. In contrast, insulin use has an inverse association with NPC mortality. This may be because patients who inject insulin to control diabetes have a worse blood glucose status, often accompanied by serious complications. In addition, insulin is regarded as a promoting factor in cancer development in some studies [20].

Chemotherapy has been shown to be related to a higher cancer-specific mortality rate. NCCN guidelines state that chemotherapy can be given at a more advanced stage [21]. This may explain why chemotherapy has been shown to be related to a higher cancer-specific mortality rate. Chronic kidney disease and heart failure have also been shown to be related to a higher cancer-specific mortality rate. Since CKD and heart failure patients cannot tolerate many chemotherapy regimens, this may explain why these patients had worse survival.

We further investigated the combination effect of Metformin with AGI and DPP4 inhibitors in Table 4. Metformin with AGI had a low HR. Metformin with DPP4 inhibitors also had a low HR; however, the result was not statistically significant. This may be due to the low number of patients taking both metformin and DPP4 inhibitors. We aim to conduct this study again when more patient data are available.

A limitation of this study is that we did not analyze patients receiving SGLT-2 inhibitors and GLP-1 agonists. Our data are till 2017, and the SGLT-2 inhibitor and GLP-1 agonists were not frequently used at that time. The mechanism of GLP-1 agonists is similar to that of DPP4 inhibitors, and the SGLT-2 inhibitor has been seen to suppress cancer development in vitro [22]. Also, this study is a retrospective cohort study, and as such, it is possible that confounding factors may exist. To mitigate this bias, we have taken steps to consider factors such as age, sex, medication (Sulphonylurea, Thiazolidinediones, AGI, Insulin, DPP4 inhibitors and Meglitinides), treatments (Radiation therapy and Chemotherapy), and comorbidities (hypertension, hyperlipidemia, chronic obstructive pulmonary disease, chronic kidney disease, and heart failure). Moreover, we acknowledge the opportunity for further investigation into the drugs used in chemotherapy. The standard chemotherapy regimen for concurrent chemoradiotherapy in nasopharyngeal cancer involves cisplatin, while the standard induction chemotherapy regimens include TPF (docetaxel, cisplatin, and 5-FU) or GC (gemcitabine and cisplatin) [23]. The MEPFL regimen (mitomycin C, epirubicin, cisplatin, and 5-fluorouracil/leucovorin) was also used in Taiwan [24]. We also intend to expand our research to include more information about radiotherapy. In the past year, 3D-conformal Radiation Therapy (3D-CRT) has been a popular radiation technique. It is well-known that Intensity-Modulated Radiation Therapy (IMRT) has become increasingly popular in recent years, with more and more trials focusing on its use [25]. In Taiwan, proton therapy has recently been implemented [26]. Our research aims to explore the continued effectiveness of metformin for patients undergoing both IMRT and proton therapy. We also seek to investigate whether a dose-response relationship exists between radiation dose and reduced mortality rates in NPC patients taking metformin. Moreover, we are interested in understanding the impact of the sequence of radiotherapy and chemotherapy on the treatment outcomes of NPC patients taking metformin. Specifically, we want to investigate whether concurrent chemoradiotherapy or sequential treatment leads to better outcomes for NPC patients taking metformin. We will continuously update our findings as we gather new data.

## 5. Conclusions

Metformin may be effective at reducing the cancer-specific mortality rate in nasopharyngeal cancer patients. Further randomized control trials should be undertaken.

## Figures and Tables

**Table 1:** Characteristics of nasopharyngeal cancer patients receiving Metformin versus not receiving Metformin.

	Metformin				
	No *n* = 3039		Yes *n* = 3039		
Variable	*n*	(%)	*n*	(%)	*p*-Value
Sex					0.56
Female	783	25.8	763	25.1	
Male	2256	74.2	2276	74.9	
Age group (years)					0.34
20–49	444	14.6	485	16.0	
50–65	1549	51.0	1524	50.2	
>65	1046	34.4	1030	33.9	
Age (years), mean ± standard deviation	60.4 ± 10.4		59.9 ± 10.5		0.07
Medications					
Sulphonylurea	1347	44.3	2341	77.0	<0.001
Thiazolidinediones	314	10.3	501	16.5	<0.001
AGI	402	13.2	688	22.6	<0.001
Insulin	1842	60.6	2324	76.5	<0.001
DPP4 inhibitors	708	23.3	1039	34.2	<0.001
Meglitinides	425	14.0	711	23.4	<0.001
Comorbidities					
Hypertension	2077	68.3	2006	66.0	0.05
Hyperlipidemia	1954	64.3	1676	55.2	<0.001
Chronic obstructive pulmonary disease	747	24.6	721	23.7	0.44
Chronic kidney disease	163	5.36	67	2.20	0.001
Heart failure	159	5.23	126	4.15	0.045
Treatment					
Radiation therapy	2608	85.8	2333	76.8	<0.001
Chemotherapy	2176	71.6	1927	63.4	<0.001

**Table 2:** Incidence density of deaths due to nasopharyngeal cancer between Metformin use group and non-Metformin use group.

	Metformin No			Metformin Yes				
Variable	Event	Person- Years	Incidence Rate	Event	Person- Years	Incidence Rate	Crude HR (95% CI)	Adjusted HR (95% CI) ^†^
All	662	12,417	5.33	661	16,484	4.01	0.81 (0.73, 0.91) ***	0.80 (0.71, 0.90) ***
Sex								
Female	153	3781	4.05	133	4459	2.98	0.78 (0.62, 0.99) *	0.80 (0.62, 1.04)
Male	509	8636	5.89	528	12,025	4.39	0.81 (0.72, 0.92) ***	0.79 (0.69, 0.91) ***
Age group (years)								
20–49	76	2732	2.78	107	3288	3.25	1.19 (0.89, 1.61)	0.99 (0.70, 1.41)
50–65	328	6581	4.98	320	8927	3.58	0.77 (0.66, 0.90) **	0.72 (0.61, 0.86) ***
>65	258	3105	8.31	234	4269	5.48	0.74 (0.62, 0.89) **	0.80 (0.66, 0.96) *
Medications								
Sulphonylurea								
No	316	8109	3.90	163	3024	5.39	1.31 (1.08, 1.58) **	1.13 (0.92, 1.38)
Yes	346	4308	8.03	498	13,460	3.70	0.55 (0.47, 0.63) ***	0.63 (0.54, 0.72) ***
Thiazolidinediones								
No	575	11,223	5.12	556	12,625	4.40	0.90 (0.80, 1.01)	0.82 (0.72, 0.94) **
Yes	87	1195	7.28	105	3858	2.72	0.45 (0.33, 0.60) ***	0.51 (0.38, 0.70) ***
AGI								
No	562	10,938	5.14	524	11,558	4.53	0.92 (0.81, 1.03)	0.83 (0.73, 0.95) **
Yes	100	1479	6.76	137	4926	2.78	0.51 (0.39, 0.66) ***	0.62 (0.47, 0.83) **
Insulin								
No	152	4871	3.12	97	3809	2.55	0.89 (0.69, 1.14)	0.97 (0.73, 1.29)
Yes	510	7546	6.76	564	12,675	4.45	0.71 (0.63, 0.80) ***	0.78 (0.68, 0.89) ***
DPP4 inhibitors								
No	500	10,061	4.97	482	9112	5.29	1.06 (0.94, 1.20)	0.88 (0.76, 1.01)
Yes	162	2356	6.88	179	7372	2.43	0.45 (0.37, 0.57)***	0.54 (0.43, 0.68) ***
Meglitinides								
No	540	11,089	4.87	502	11,868	4.23	0.91 (0.81, 1.03)	0.83 (0.73, 0.95) **
Yes	122	1328	9.19	159	4615	3.45	0.46 (0.36, 0.58) ***	0.60 (0.47, 0.78) ***
Treatment								
Radiation therapy								
No	84	3239	2.59	104	4335	2.40	0.86 (0.64, 1.14)	0.68 (0.47, 1.00)
Yes	578	9179	6.30	557	12,149	4.58	0.82 (0.73, 0.92) ***	0.80 (0.70, 0.91) ***
Chemotherapy								
No	155	4949	3.13	156	6775	2.30	0.74 (0.59, 0.93) **	0.79 (0.60, 1.03)
Yes	507	7468	6.79	505	9709	5.20	0.86 (0.76, 0.97) *	0.79 (0.69, 0.91) ***
Comorbidity								
No	82	3246	2.53	109	3044	3.58	1.37 (1.02, 1.82) *	1.21 (0.83, 1.75)
Yes	580	9171	6.32	552	13,440	4.11	0.74 (0.66, 0.83) ***	0.73 (0.64, 0.83) ***

Incidence rate: 100 person-years; HR: hazard ratio. ^†^ Adjusted for sex, age, medications (Sulphonylurea, thiazolidinediones, AGI, insulin, DPP4, meglitinides), treatment (radiation therapy, chemotherapy), and comorbidities of hypertension, hyperlipidemia, chronic obstructive pulmonary disease, chronic kidney disease, and heart failure. * *p*-value < 0.05, ** *p*-value < 0.01, *** *p*-value < 0.001.

**Table 3:** Hazard ratio and 95% confidence interval of death due to nasopharyngeal cancer associated with medications, treatment, and co-variables.

	Crude			Adjusted ^†^		
Variable	HR	(95% CI)	*p*-Value	HR	(95% CI)	*p*-Value
Sex (male vs. female)	1.37	(1.20, 1.56)	<0.001	1.45	(1.27, 1.65)	<0.001
Age (every year)	1.03	(1.02, 1.03)	<0.001	1.03	(1.02, 1.03)	<0.001
Metformin use (nonuse as a control)	0.81	(0.73, 0.91)	<0.001	0.80	(0.71, 0.90)	<0.001
Medications						
Sulphonylurea	1.12	(1.00, 1.25)	0.049	1.10	(0.96, 1.25)	0.16
Thiazolidinediones	0.88	(0.75, 1.03)	0.10	0.88	(0.75, 1.04)	0.13
AGI	0.83	(0.72, 0.96)	0.01	0.83	(0.71, 0.97)	0.02
Insulin	1.95	(1.70, 2.24)	<0.001	2.16	(1.87, 2.50)	<0.001
DPP4 inhibitors	0.72	(0.64, 0.82)	<0.001	0.64	(0.56, 0.74)	<0.001
Meglitinides	1.09	(0.95, 1.24)	0.23	1.07	(0.92, 1.23)	0.39
Treatment						
Radiation therapy	1.82	(1.56, 2.13)	<0.001	1.20	(0.99, 1.45)	0.07
Chemotherapy	1.95	(1.71, 2.21)	<0.001	2.03	(1.73, 2.38)	<0.001
Comorbidity						
Hypertension	1.39	(1.24, 1.57)	<0.001	1.16	(1.02, 1.31)	0.03
Hyperlipidemia	1.22	(1.09, 1.36)	<0.001	1.12	(0.99, 1.26)	0.07
Chronic obstructive pulmonary disease	1.18	(1.04, 1.33)	0.01	0.96	(0.84, 1.09)	0.51
Chronic kidney disease	2.23	(1.74, 2.86)	<0.001	1.58	(1.22, 2.03)	<0.001
Heart failure	1.93	(1.53, 2.43)	<0.001	1.68	(1.32, 2.13)	<0.001

^†^ Adjusted for sex, age, medications (Sulphonylurea, thiazolidinediones, AGI, insulin, DPP4, meglitinides), treatment (radiation therapy, chemotherapy), and comorbidities of hypertension, hyperlipidemia, chronic obstructive pulmonary disease, chronic kidney disease, and heart failure.

**Table 4:** Cox proportional hazard regression analysis for the risk of death due to nasopharyngeal cancer-associated metformin use with the combined effect of AGI.

Variable		Event	Person- Years	Incidence Rate	Crude HR (95% CI)	Adjusted HR (95% CI) ^†^
Metformin	AGI					
No	No	562	10,938	5.14	1 (Reference)	1 (Reference)
No	Yes	100	1479	6.76	1.25 (1.01, 1.55) *	1.05 (0.83, 1.31)
Yes	No	524	11,558	4.53	0.92 (0.81, 1.03)	0.86 (0.75, 0.98) *
Yes	Yes	137	4926	2.78	0.63 (0.52,0.76) ***	0.62 (0.50,0.76) ***
Metformin	DPP4					
No	No	500	10,061	4.97	1 (Reference)	1 (Reference)
No	Yes	162	2356	6.88	1.23 (1.03, 1.47) *	0.88 (0.73, 1.07)
Yes	No	482	9112	5.29	1.06 (0.94, 1.21)	0.93 (0.81, 1.07)
Yes	Yes	179	7372	2.43	0.55(0.46, 0.65) ***	0.48 (0.40, 1.03)

Incidence rate: 100 person-years; HR: hazard ratio; DPP4: DPP4 inhibitors. ^†^ Adjusted for sex, age, medications (Sulphonylurea, thiazolidinediones, AGI, insulin, DPP4, meglitinides), treatment (radiation therapy, chemotherapy), and comorbidities of hypertension, hyperlipidemia, chronic obstructive pulmonary disease, chronic kidney disease, and heart failure. * *p*-value < 0.05, *** *p*-value < 0.001.

## Data Availability

The data presented in this study are available in this article.
